# The correlation between parents' education attainment and humanistic literacy of eight-year medical students

**DOI:** 10.5116/ijme.661d.10af

**Published:** 2024-05-14

**Authors:** Yao Yu, Guiyi Yang, Yixuan Qin, Linhan Fang, Yuxuan Liao, Wenxuan Bai, Jianzhen Wu, Pengfei Rong

**Affiliations:** 1 Department of Health Management, The Third Xiangya Hospital, Central South University, Changsha, China; 2 Xiangya School of Medicine, Central South University, Changsha, China; 3 Xiangya School of Public Health, Central South University, Changsha, China; 4 National Cancer Center/National Clinical Research Center for Cancer/Cancer Hospital, Chinese Academy of Medical Sciences and Peking Union Medical College, Beijing, China; 5 Department of Radiology, The Third Xiangya Hospital, Central South University, Changsha, China

**Keywords:** Humanistic literacy, eight-year clinical medical students, parents’ education attainment, home-school collaboration, family education

## Abstract

**Objectives:**

To explore the content, ways, and methods of family education in cultivating students’ humanistic literacy.

**Methods:**

We used a cross-sectional study and collected questionnaire data from 616 eight-year clinical medical students of Central South University by a convenience sampling survey. To determine the influence of parents’ educational attainment on children’s humanistic literacy, the students were mainly divided into two groups including parents’ education attainment was college or above (Group B) and parents’ education attainment below college (Group A). Non-parametric tests are used to test the differences between the two groups in humanistic spirit, interpersonal communication, humanistic knowledge and ability, and development planning.

**Results:**

Group B had better social morality and a sense of social responsibility than group A (P=0.024, P=0.001). Compared to group A, students in group B could better integrate into the new environment, communicate with students from different institutes, and take an active part in activities (P=0.001). In a nutshell, students in group B had more excellent humanistic knowledge and ability and could consult medical literature and write in Chinese or English more proficiently than group A (P=0.0001, P=0.0001).

**Conclusions:**

We found that the eight-year medical students whose parents’ highest education attainment is college or above almost mastered a higher level of humanistic literacy. It demonstrated family humanistic literacy education is irreplaceable. We recommend systematic efforts to build a reasonable and effective family humanistic literacy education platform and form an educational synergy with school education to make the cultivation of humanistic literacy among students more efficient.

## Introduction

Humanistic literacy refers to people’s comprehensive qualities in terms of humanistic spirit, interpersonal communication, humanistic knowledge and ability, development planning, etc. In the medical field, it also emphasizes the specific embodiment of individual culture and internal character of medical staff in medical work, such as empathy ability, communication ability, and moral reasoning.^1^^,^^2^ With the improvement in the living standards of people, the demand for medical services is rapidly increasing. Healthcare is undergoing a paradigm shift toward providing comfortable and safe medical services.^3^ Therefore, the continuous development of medical technology and professional knowledge cannot completely solve the problems in contemporary medical services. For example, the disharmonious doctor-patient relationship still exists in China.^4^^,^^5^ In addition to the imperfect medical security system and patients’ cognitive differences, the other main reason is existing insufficient communication skills and humanistic care among medical staff. Multiple factors need to be considered for the humanistic literacy of eight-year medical students is uneven. Two main reasons can account for this issue properly as follows. For one thing, defective knowledge structure and heavy professional courses cause eight-year program medical students to pay less attention to humanistic literacy.^6^^,^^7^ Part of the eight-year program medical students always regard humanities courses as elective courses or auxiliary courses and the purpose of learning humanities courses is only to obtain credits.^8^ As a result, the humanistic literacy of some eight-year program medical students declines obviously. For another thing, the difference in parents' educational level and growth environment bring out significant humanistic literacy differences between eight-year program medical students.^9^ However, the importance of family education is often overlooked.^10^ Correlational research has shown that compared with less educated parents, those who are more educated can create a harmonious family atmosphere and pay more attention to accompanying their children as they grow up and show a tendency to behave more respectfully, comprehensively, and patiently to their children. Meanwhile, more educated parents would like to give comfort, encouragement, and sympathy when their children make mistakes so that in the future their children will be more confident, friendly, and kind in interpersonal communication.^11^^,^^12^

Eight-year medical students as the future medical industry high-level talents, the significance of cultivating their humanistic literacy is self-evident. Therefore, it’s inevitable to deepen the eight-year program medical students’ understanding of humanistic literacy and enhance their humanistic care ability when cultivating excellent doctors and developing medical sciences.^13^ To our knowledge, nowadays most research on the humanistic literacy of eight-year medical students focuses on humanities courses and education methods in their training program and few of them have specifically analyzed the influence of parental education level on child humanistic literacy.

This paper aims to explore the content, ways, and methods of family education in cultivating students’ humanistic literacy, build a reasonable and effective family humanistic literacy education platform, and form an educational synergy with school education, thereby continuously improving the humanistic literacy of eight-year medical students.

## Method

This study was performed in accordance with the Declaration of Helsinki. The study and all the methods were approved by the Third Xiangya Hospital Ethics Affiliation of Central South University. All participants in the study carefully read and signed the informed consent.

### Study Design

We used a cross-sectional study and aimed to investigate the influence of parents’ education attainment on the humanistic literacy of eight-year clinical medical students of Central South University. This questionnaire was composed of five parts, one was the basic information, and the other four were humanistic literacy assessments, using the 5-point Likert scale. Their attitudes were graded as follows: (1) totally disagree; (2) disagree; (3) uncertain; (4) agree; (5) totally agree.

The first block covered demographic questions including gender, grade (including freshman, Sophomore, the third year of college until the eighth year of college), nationality (including the Han nationality and Non-han nationality), born city (including Provincial capital or municipality, prefecture-level city, County-level cities, Sub-county cities or rural). Besides, it also included their parents’ information, such as occupations, (including Superintendents of government agencies, party and mass; organizations, enterprises, and public institutions; Professional and technical personnel; Office staff and related personnel; Business and service industry personnel; Production personnel in agriculture, forestry, animal husbandry, fishery and water conservancy industries; Production and transportation equipment operators and related personnel; Military personnel; Other practitioners who are inconvenient to classify), and education level.

The second block demonstrated eight-year students’ self-evaluation of humanistic spirit. There were 5 questions, including I can maintain public order and resist immoral behaviors with patriotism; I have national self-esteem, national self-confidence, and a sense of social responsibility; I have a correct dialectical understanding of Chinese traditional culture that means “Take the essence and discard the dross”(Ma NC, “Yanshan Night Talk” 25) and different culture with cultural inclusion; I advocate human-oriented, human dignity and unlimited care for people; I can establish a correct outlook on life and values and handle social and personal relationships properly.

The third block evaluated their interpersonal communication, including that I can handle love relationships properly and deal well with interpersonal relationships; I can integrate into the new environment, communicate with students from different institutes, and take an active part in activities; I cannot express inner feelings, causing abnormal behaviors.

The fourth block showed their humanistic knowledge and ability. The details were as follows: I possess knowledge of history, literature, politics, law, art, philosophy, religion, morality, and language; I can consult medical literature and write in Chinese or English proficiently; I have the skills to grasp the macro situation such as organizing events and coordinating the relations; I can conduct an accurate analysis of existing information and make comparative judgments; I can accept new things with unique and dialectical insights and solve problems with strong innovative thinking; I have a rich imagination, strong observation, and intuitive understanding.

The last block estimated their development planning including 4 questions: I can develop interest, clarify aspirations, make career plans, and survive in society; I think the learning experience in the old campus where general education is received is good for the future study, life and research; I can persistently pursue my major and correct learning attitude with love for medicine; I can't deal well with survival pressure with an ambiguous developmental direction.

### Data Collection and Participants

The questionnaires were distributed in November 2014 through a platform named Wenjuanxing. All participants from Xiangya Medical School of Central South University major in eight-year clinical medicine. They receive eight years of medical education in school, do not participate in internships, are not residents, and finally receive a professional doctorate in clinical medicine. In addition, all participants in the study carefully read and signed the informed consent.

At last, we collected 650 questionnaires in total, of which 616 had effective and exhaustive responses. The content of the questionnaire was reviewed by an expert panel, including a medical education specialist, a student affairs management officer responsible for the clinical undergraduates’ training programs, an associate professor of health statistics, an associate professor of the epidemiological specialist, and an expert in humanistic literacy. We also pretested the survey with 30 students and revised the questionnaire according to the feedback. Cronbach’s Alpha of the total scale in this research (Alpha=0.877) was examined for testing the internal consistency reliability.

### Data Analysis

The purpose of the study was to figure out the influence of parents’ education attainment on children’s humanistic literacy. Accordingly, we divided the participants into two groups based on their parents’ educational attainment. Participants whose parents’ education attainment was college or above belonged to group B, while others belonged to group A. It should be noted that we chose the parent with higher education attainment as the grouping criterion.

We first used the Kolmogorov-Smirnov test to analyze the data distribution and found it presented a skewed distribution. So, we applied non-parametric tests to test the differences between the two groups, including humanistic spirit, interpersonal communication, humanistic knowledge and ability, and development planning, with 18 questions in total. The chi-square test and the nonparametric test were respectively used in basic characteristics and humanistic literacy assessments for comparing the constituent ratios of scores 1-5 between groups A and B in every question. All the analyses above were done with SPSS.27.0.1 and a p-value of <0.05 was considered statistically significant.

## Results

### Demographic Characteristics and Basic Information

A total of 616 valid questionnaires were collected, including 326 questionnaires in which parents’ education attainment is college or above (group B), and 290 questionnaires about parents’ education attainment is below college (group A). There were 154 (53.1%) men and 136 (46.9%) women in group A. In group B, 160 (49.1%) were men and 166 (50.9%) were women. The proportion was similar between the two groups in grade composition. The number of freshmen and sophomores was 119 (41.0%) and 149 (45.1%) respectively. Compared with group B, group A had a significant difference in their city of birth, the latter students mainly came from the sub-county cities (χ^2^(3, N=616)=117.44, P=0.0001). There was no statistical significance in nationality composition. Most of the students were Han nationality in both groups. While a clear difference could be found in the father’s profession. The majority of fathers worked in professional and technical jobs in group B (χ^2^(14,N = 616) =226.69, P=0.0001). However, the proletarian labor force was the majority in group A (178, 61.4%). The same situation could also be seen in the mother’s profession. The basic characteristics of the participants were presented in Table 1.

### Subjective Cognition in Humanistic Spirit

To compare the subjective cognition of eight-year students in humanistic spirit, a 5-point Likert scale with 5 questions was used to evaluate. Compared to group A, students in group B were more patriotic and had better social morality (χ^2^(1, N=616) =5.11, P=0.024). They also had stronger national self-esteem, national self-confidence, and a sense of social responsibility (χ^2^(1, N=616)=12.05,P=0.001). Nearly 90% of students of group A (255,88.0%) and group B (295,90.5%) had a correct and dialectical understanding of Chinese traditional culture and different cultures with cultural inclusion (χ^2^(1, N=616)=6.85, P=0.009). Besides, we observed that 86.6% (251) of group A and 90.5% (295) of group B advocated human-oriented, human dignity and unlimited care for people (χ^2^(1, N=616) =4.27, P=0.039). No significant differences were found in the following question: I can establish a correct outlook on life and values and handle social and personal relationships properly. The specific data was revealed in Table 2.

### Subjective Cognition in Interpersonal Communication

Interpersonal communication is a crucial topic for clinical treatment, so it is of vital importance for eight-year medical students to master interpersonal communication abilities. To estimate the abilities of the two groups, three queries were used to score them with a 5-point Likert scale. The results showed that 4.5% more people in group B (243,74.5%) than in group A (203,70.0%) can handle love relationships properly and deal well with interpersonal relationships (χ^2^(1, N=616)=6.44, P=0.011).

**Table 1:** Demographic characteristics and basic information of eight-year medical

Basic characteristics		Group A	Group B	P
		(n = 290) (%)	(n = 326) (%)	
Gender				
	Male	154(53.1)	160(49.1)	0.359
	Female	136(46.9)	166(50.9)	
Grade				
	College freshman and Sophomore	119(41.0)	147(45.1)	0.351
	From third year of college to eighth year of college	171(59.0)	179(54.9)	
City of birth				
	Provincial capital or municipality	22(7.6)	83(25.5)	0.0001***
	Prefecture-level city	51(17.6)	116(35.6)	
	County-level cities	73(25.2)	86(26.4)	
	Sub-county cities	144(49.7)	41(12.6)	
Nationality				
	Han nationality	280(96.6)	302(92.6)	0.052
	Non-Han nationality	10(3.4)	24(7.4)	
Father's profession				
	Superintendents of government agencies, party, and mass organizations, enterprises, and public institutions	17(5.9)	48(14.7)	0.0001***
	Professional and technical personnel	42(14.5)	148(45.4)	
	Office staff and related personnel	8(2.8)	43(13.2)	
	Business and service industry personnel	78(26.9)	13(4)	
	Production personnel in agriculture, forestry, animal husbandry, fishery and water conservancy industries	60(20.7)	5(1.5)	
	Production and transportation equipment operators and related personnel	46(15.9)	25(7.7)	
	Military personnel	2(0.6)	2(0.6)	
	Other practitioners who are inconvenient to classify	37(12.7)	42(12.9)	
Mother's profession				
	Superintendents of government agencies, party and mass organizations, enterprises, and public institutions	8(2.8)	30(9.2)	0.0001***
	Professional and technical personnel	29(10)	155(47.5)	
	Office staff and related personnel	5(1.7)	21(6.4)	
	Business and service industry personnel	75(25.9)	14(4.3)	
	Production personnel in agriculture, forestry, animal husbandry, fishery and water conservancy industries	67(23.1)	4(1.2)	
	Production and transportation equipment operators and related personnel	44(15.2)	27(8.3)	
	Military personnel	0(0)	1(0.3)	
	Other practitioners who are inconvenient to classify	62(21.4)	74(22.7)	

The chi-square test was used for comparing the basic characteristics of the groups. P < 0.05 was indicated in bold. *P < 0.05, **P < 0.01, ***P < 0. 001.Group A (n1 = 290) and group B (n2 = 326) were included.

**Table 2:** Comparison of the subjective cognition in humanistic spirit of eight-year medical students

Basic characteristics		Group A	Group B	P
		(n = 290) (%)	(n = 326) (%)	
I can maintain public order and resist immoral behaviors with patriotism				
	Totally disagree	1(0.3)	3(0.9)	0.024*
	Disagree	2(0.7)	3(0.9)	
	Uncertain	17(5.9)	15(4.6)	
	Agree	71(24.5)	53(16.3)	
	Totally agree	199(68.6)	252(77.3)	
I have national self-esteem, national self-confidence and sense of social responsibility				
	Totally disagree	1(0.3)	2(0.6)	0.001**
	Disagree	3(1)	5(1.5)	
	Uncertain	20(6.9)	12(3.7)	
	Agree	81(27.9)	56(17.2)	
	Totally agree	185(63.9)	251(77.0)	
I have a correct and dialectical understanding of Chinese traditional culture and different culture with cultural inclusion				
	Totally disagree	0(0.0)	2(0.6)	0.009**
	Disagree	3(1.0)	7(2.1)	
	Uncertain	32(11.0)	22(6.8)	
	Agree	91(31.4)	75(23.0)	
	Totally agree	164(56.6)	220(67.5)	
I advocate human-oriented, human dignity and unlimited care for people				
	Totally disagree	1(0.3)	0(0.0)	0.039*
	Disagree	2(0.7)	4(1.2)	
	Uncertain	36(12.4)	27(8.3)	
	Agree	84(29.0)	82(25.2)	
	Totally agree	167(57.6)	213(65.3)	
I can establish a correct outlook on life and values and handle social and personal relationships properly				
	Totally disagree	1(0.3)	1(0.3)	0.136
	Disagree	3(1)	5(1.5)	
	Uncertain	22(7.6)	22(6.8)	
	Agree	95(32.8)	87(26.7)	
	Totally agree	169(58.3)	211(64.7)	

The nonparametric test was used for the comparison. P < 0.05 was indicated in bold. *P < 0.05, **P < 0.01. Group A (n1 = 290) and group B (n2 = 326) were included.

Meanwhile, students in group B (225,69.0%) had more exceptional adaptability and communication skills than group A (173,59.7%). They could integrate into the new environment, communicate with students from different institutes, and take an active part in activities (χ^2^(1, N = 616) =10.66, P=0.001). Particularly, most of the students in both groups (group A: 76.8% (223) and group B: 78.8% (257)) disagreed or totally disagreed about the question that couldn't express inner feelings, causing abnormal behaviors, with no statistical significance discovered. Table 3 tabulated the concrete contents.

### Subjective Cognition in Humanistic Knowledge and Ability

Humanistic knowledge and ability are an integral part of humanistic literacy for eight-year medical students. Accordingly, we compared differences between the two groups through six questions, which demonstrated a high degree of diversity (Table 4). In a nutshell, students in group B had more excellent humanistic knowledge and ability levels than group A. Most students in group B (235,72.1%) possessed knowledge of history, literature, politics, law, art, philosophy, religion, morality, and language (χ^2^(1, N=616)=20.08, P=0.0001). They could as well consult medical literature and write in Chinese or English proficiently, which was better than 16.2% of group A (χ^2^(1, N=616)=20.21, P=0.0001). 59.6% (173) of group A and 66.3% (216) of group B expressed that they had the skills to grasp the macro situation such as organizing events and coordinating the relations (χ^2^(1, N = 616)=6.89, P=0.009).

**Table 3:** Comparison of the subjective cognition in interpersonal communication of eight-year medical students

Basic characteristics		Group A	Group B	P
		(n = 290) (%)	(n = 326) (%)	
I can handle love relationship properly and deal well with interpersonal relationship				
	Totally disagree	1(0.3%)	4(1.2%)	0.011*
	Disagree	10(3.5%)	13(4.0%)	
	Uncertain	76(26.2%)	66(20.3%)	
	Agree	116(40.0%)	105(32.2%)	
	Totally agree	87(30.0%)	138(42.3%)	
I can integrate into the new environment, communicate with students from different institutes, and take an active part in activities				
	Totally disagree	6(2.0%)	10(3.1%)	0.001**
	Disagree	22(7.6%)	32(9.8%)	
	Uncertain	89(30.7%)	59(18.1%)	
	Agree	109(37.6%)	101(31.0%)	
	Totally agree	64(22.1%)	124(38.0%)	
I can't express inner feelings, causing abnormal behaviors				
	Totally disagree	155(53.4%)	196(60.1%)	0.211
	Disagree	68(23.4%)	61(18.7%)	
	Uncertain	33(11.4%)	25(7.7%)	
	Agree	19(6.6%)	20(6.1%)	
	Totally agree	15(5.2%)	24(7.4%)	

The nonparametric test was used for the comparison. P < 0.05 was indicated in bold. *P< 0.05, **P < 0.01. Group A (n1 = 290) and group B (n2 = 326) were included.

**Table 4:** Comparison of the subjective cognition in humanistic knowledge and ability of eight-year medical students

Basic characteristics		Group A	Group B	P
		(n = 290) (%)	(n = 326) (%)	
I possess knowledge of history, literature, politics, law, art, philosophy, religion, morality and language				
	Totally disagree	3(1.0)	5(1.5)	0.0001***
	Disagree	20(6.9)	8(2.5)	
	Uncertain	94(32.4)	78(23.9)	
	Agree	116(40.0)	120(36.8)	
	Totally agree	57(19.7)	115(35.3)	
I can consult medical literature and write in Chinese or English proficiently				
	Totally disagree	9(3.1)	2(0.6)	0.0001***
	Disagree	29(10.0)	32(9.8)	
	Uncertain	129(44.5)	101(31.0)	
	Agree	90(31.0)	108(33.1)	
	Totally agree	33(11.4)	83(25.5)	
I have the skills to grasp the macro situation such as organizing events and coordinating the relations				
	Totally disagree	0(0.0)	1(0.3)	0.009**
	Disagree	15(5.2)	13(4.0)	
	Uncertain	102(35.2)	96(29.4)	
	Agree	114(39.3)	115(35.3)	
	Totally agree	59(20.3)	101(31.0)	
I can conduct accurate analysis of existing information and make comparative judgments				
	Totally disagree	1(0.3)	2(0.6)	0.01*
	Disagree	3(1.1)	4(1.2)	
	Uncertain	63(21.7)	55(16.9)	
	Agree	145(50.0)	143(43.9)	
	Totally agree	78(26.9)	122(37.4)	
I can accept new things with unique and dialectical insights and solve problems with strong innovative thinking				
	Totally disagree	0(0.0)	0(0.0)	0.001**
	Disagree	8(2.8)	12(3.7)	
	Uncertain	88(30.3)	74(22.7)	
	Agree	134(46.2)	126(38.6)	
	Totally agree	60(20.7)	114(35.0)	
I have a rich imagination, strong observation and intuitive understanding				
	Totally disagree	2(0.7)	1(0.3)	0.0001***
	Disagree	13(4.5)	8(2.5)	
	Uncertain	81(27.9)	79(24.2)	
	Agree	131(45.2)	111(34)	
	Totally agree	63(21.7)	127(39)	

The nonparametric test was used for the comparison. P < 0.05 was indicated in bold. *P<0.05, **P < 0.01, ***P < 0. 001.Group A (n1 = 290) and group B (n2 = 326) were included.

At the same time, we observed that students in group B (265,81.3%) could conduct a more accurate analysis of existing information and make comparative judgments compared with group A (223,76.9%) (^χ^2(1, N= 616) =6.66, P=0.01). Similarly, 73.6% (240) of group B could accept new things with unique and dialectical insights and solve problems with strong innovative thinking, 6.7% more than group A (194,66.9%) (χ^2^(1, N = 616) =10.77, P=0.001). As for the question: I have a rich imagination, strong observation, and intuitive understanding, there was an extremely significant statistical difference (χ^2^(1, N = 616) =14.53, P=0.0001).

### Subjective Cognition in Development Planning

Finally, we investigated their plans for the future (Table 5). More than 70% of students (group A: 72.4% (210) and group B: 76.7% (250)) could develop interest, clarify aspirations, make career plans, and survive in society (χ^2^(1, N= 616) =11.89, P=0.001). The same proportion (63.8%) of students in both groups thought the learning experience in the old campus was good for future study, life, and research. However, a stronger determination to medicine was manifested in group B (296,90.8%). They could persistently pursue their major and correct learning attitude with love for medicine (χ^2^(1, N= 616) =6.53, P=0.011). Fortunately, most students in group B (251,77.0%) did not have the problem of cannot deal well with survival pressure with an ambiguous developmental direction (χ^2^(1, N= 616)=7.03, P=0.008).

## Discussion

Medical education is an eternal pursuit of cultivating a humanistic spirit and superb skills of clinical medical interdisciplinary talents.^14^ Improving the humanistic literacy of medical students is inseparable from humanistic literacy education. Strengthening the humanistic literacy education of students and establishing a correct concept of humanistic literacy education will help alleviate the problem of doctor-patient tension at the present stage.^15^ However, many reasons lead to medical humanistic literacy education is not in place. With the rapid development of China’s economy, under the influence of the whole society’s bad ideas such as more emphasis on science, engineering, agriculture, and medicine than literature, professional skills than comprehensive quality, clinical than foundation, and the mentality of eagerness for quick success and instant benefit, the cultivation of comprehensive quality still falls on the professional technical ability.16 This is not conducive to the development of humanistic literacy.

Our research results showed that parents’ educational attainment also made a difference in students’ humanistic literacy via conducting a questionnaire survey on humanistic literacy of eight-year clinic medical students of Central South University. Generally speaking, students whose parents had higher education attainment demonstrated a stronger ability in humanistic literacy, including political literacy, interpersonal communication, knowledge level, innovation ability, and development planning. According to this phenomenon, we analyzed potential reasons. Some suggestions on ways to enhance medical students’ humanistic literacy were put forward combined with our research results.

The reason why the educational attainment of the parents affected the humanistic literacy of the students may be related to many factors. One of the factors is the time that parents spend with their children. Research has shown that in different countries around the world, better-educated parents spend more time with their children than less-educated parents.^17^^,^^18^ Children who are accompanied by their parents tend to be more fulfilled and confident in their hearts, and they often show more courage when encountering things, which is beneficial to deal with the survival pressure in the future and formulate appropriate development planning. Secondly, in a general way, parents with higher educational attainment have higher humanistic literacy. The level of parents' humanistic literacy often affects the level of family humanistic literacy education. Family humanistic literacy education plays a very important role in the formation of students’ spiritual character. The high humanistic literacy of parents has a profound appeal to students’ behavioral health; on the contrary, the lack of humanistic literacy of parents will affect the healthy development of students, producing hindrances.^19^ Moreover, parenting style was also affected by their educational attainment. Parents respect and understand their children, they are emotionally warm, and strict requirements have a positive impact on their children’s interpersonal skills.^15^ In addition, less educated parents are largely unaware of how to control their emotions in front of their children. While research indicated that children's negative effect was positively related to parental emotional discouragement for parents with generalized anxiety disorder, so that students had negative attitudes and low moods when dealing with troubles.^20^ Another aspect of the reason may be that parents have different aspirations for their children. Parents with strong educational values (i.e., belief in the importance of education) are more likely to have high-achieving children than parents with less strong educational values.^21^ This feature may be reflected in the difference in educational resources. Parents with high education levels are more likely to have the educational experience and resources to draw upon when cultivating their children to achieve a college- or graduate-level education. Parental educational aspirations for their children increased as a function of parental education. Significantly higher parental aspirations were associated with higher levels of children’s academic performance.^22^

In summary, parents’ education attainment has a great influence on children’s humanistic literacy. Family education should run through all ages and time points of children, including school time and winter and summer vacation time at home. Therefore, parents should raise awareness of the importance of humanistic literacy education in family education and should not rely entirely on the school's education of their children.

**Table 5:** Comparison of the subjective cognition in development planning of eight-year medical students

Basic characteristics		Group A	Group B	P
		(n = 290) (%)	(n = 326) (%)	
I can develop interest, clarify aspirations, make career plans, and survive in society				
	Totally disagree	1(0.3)	1(0.3)	0.001**
	Disagree	8(2.8)	13(4.0)	
	Uncertain	71(24.5)	62(19.0)	
	Agree	136(46.9)	111(34.1)	
	Totally agree	74(25.5)	139(42.6)	
I think the learning experience in old campus is good for the future study, life and research				
	Totally disagree	3(1.0)	15(4.6)	0.93
	Disagree	31(10.7)	20(6.1)	
	Uncertain	71(24.5)	83(25.5)	
	Agree	91(31.4)	104(31.9)	
	Totally agree	94(32.4)	104(31.9)	
I can persistently pursue my major and correct learning attitude with love for medicine				
	Totally disagree	2(0.7)	2(0.6)	0.011*
	Disagree	5(1.7)	1(0.3)	
	Uncertain	38(13.1)	27(8.3)	
	Agree	91(31.4)	94(28.8)	
	Totally agree	154(53.1)	202(62.0)	
I can't deal well with survival pressure with an ambiguous developmental direction				
	Totally disagree	117(40.3)	172(52.8)	0.008**
	Disagree	91(31.4)	79(24.2)	
	Uncertain	48(16.6)	39(12.0)	
	Agree	18(6.2)	17(5.2)	
	Totally agree	16(5.5)	19(5.8)	

The nonparametric test was used for the comparison. P < 0.05 was indicated in bold. *P < 0.05, **P < 0.01. Group A (n1 = 290) and group B (n2 = 326) were included.

Schools should actively build a platform for humanistic literacy education for students, invite parents to actively participate in it, and provide parents with knowledge and content related to humanistic literacy education. For instance, recommend watching cultural and historical TV programs or books at home with children, and carrying out meaningful outdoor activities with children, etc., to strengthen the family atmosphere for children's inculcation. Families with good performances can be invited to share experiences. For families who have difficulties in humanistic literacy education, the school should gladly provide assistance and pass on their experience. Parents should also ardently cooperate with the school by learning about their children's learning content and psychological state at school through home school groups, school public numbers, etc., and pay attention to communication skills with their children. Families with capability can adjust the content of their family's humanistic literacy education according to the school's educational content to complement and combine with the school. In addition, parents can learn relevant knowledge and skills by going to parenting schools or family education guidance service points and adopt good parenting styles to create a harmonious family atmosphere. Besides, they should appropriately increase the time spent with their children. At the same time, start with themselves, and pay attention to their emotional control and behaviors in daily life, setting an example for their children. Last but not least, increasing emphasis on education is an important method, helping children establish correct educational values, and appropriately raising their educational aspirations for children.

There is an old saying in China: To beat iron, one needs to make oneself hard. It is of great importance for medical students to pay more attention to enhancing humanistic literacy. Interestingly, our research discovered an attractive phenomenon: the same proportion (63.8%) of students in both groups thought the learning experience in the old campus is good for future study, life, and research. The eight-year medical students at Central South University spent two years in the old campus, during which time they were taught the fundamentals of politics, language, culture and history. To a certain extent, it shows the importance of general education in schools to help improve the humanistic literacy of medical students. General education enables medical students to be inspired by philosophy, law, aesthetics, psychology, sociology, ethics, and other humanities as soon as they enter university so that they can learn about human nature and the world and develop a correct outlook on life, the world, and morality. This will lay a good humanistic foundation for the cultivation of medical ethics.^23^ Thus, students are expected to make full use of school resources to improve themselves in all aspects. While focusing on professional learning, we should strengthen the course learning of humanistic literacy.^24^^,^^25^ Besides, reading makes one wise. Taking time out of class to read while learning professional knowledge is of great benefit to the cultivation of humanistic literacy. What’s more, encourage cross-professional and cross-disciplinary exchanges, and experience the concrete manifestation of humanistic qualities in different fields. Last but not least: in clinical practice, pay attention to observing the details of the teacher’s physical examination process, and learn the skills of communication between doctors and patients. Real knowledge comes from practice, so applying what you usually learn to your actual operation can deeply understand the importance of humanistic literacy, and so on.

Many previous studies have found a robust association between parental educational attainment and children's mental health and academic achievement, often with a positive correlation.^23^^,^^26^ Focusing on medical students, our study examined the impact of parental education on the level of humanistic literacy that is essential for medical students and proposed rationalizations based on the findings and social phenomena. However, there are shortcomings in our study. First, the study population was the eight-year students at Central South University, and the amount of data was not large enough. Second, a study has found that parents’ education levels were associated with their children’s mental health, but there are different associations found through the different combinations of children’s and parents’ sexes.^27^^-^^29^ When grouping data, we chose the attainment level of the father/mother with a higher attainment level as the standard, which probably ignored the role of the father/mother in the formation of their children's humanistic literacy. In addition, the results of this questionnaire cannot avoid the subjectivity of students. We should continue to explore how to promote the cooperation between schools and parents more efficiently to motivate the comprehensive development of humanistic literacy education for medical students. Improving the level of parents' humanistic literacy education for their children will help improve the overall humanistic literacy of medical students and lay the foundation for creating a good medical environment.

## Conclusions

We found that the eight-year medical students whose parents’ highest education attainment is college or above (group B) almost mastered a higher level of humanistic spirit, interpersonal communication, humanistic knowledge and ability, and development planning. As students’ first teachers, parents provide students with the longest and most profound moral education. Compared with the systematic and macroscopic nature of school education, family education has the characteristic of focusing on the small and subtle aspects of daily life. The noble sentiments and behaviors of highly educated parents often exert a positive and lasting impact on students. On the contrary, parents who are not well educated and pay less attention to education, not only have extremely little awareness of cultivating students' humanistic literacy but also are particularly lack of methods. This will weaken humanistic literacy education in schools and directly affect students' acquisition of humanistic literacy knowledge. Therefore, to make school education more effective with half the effort, we must correctly understand the irreplaceability and irreversibility of family humanistic literacy education.

Based on these findings, we recommend systematic efforts to build a reasonable and effective family humanistic literacy education platform, form an educational synergy with school education, and continuously improve the humanistic literacy of eight-year medical students. These efforts should include parents considering further study in parenting schools or family education guidance service points and adopting good parenting styles to accompany the healthy and comprehensive growth of children. Meanwhile, building a platform for home-school cooperation and constantly improving the home-school communication mechanism will more efficiently cultivate students' humanistic literacy.

### Acknowledgements

We are greatly indebted to the eight-year medical students who completed our questionnaires. We also appreciate the team members, as well as the coordinators who assisted in questionnaire distribution and data collection. This work was supported by 2021 Central South University Course Ideological and Political Construction Research Project: “Imaging Medicine and Nuclear Medicine” Course Ideological and Political Teaching Practice and National Entrepreneurial Training Project of Central South University (Grant No. 202210533089X).

### Conflicts of Interest

The authors declare they have no conflicts of interest.
